# A dataset for Wi-Fi-based human activity recognition in line-of-sight and non-line-of-sight indoor environments

**DOI:** 10.1016/j.dib.2020.106534

**Published:** 2020-11-18

**Authors:** Baha’ A. Alsaify, Mahmoud M. Almazari, Rami Alazrai, Mohammad I. Daoud

**Affiliations:** 1 Department of Network Engineering and Security, Jordan University of Science and Technology, P.O. Box 3030, Irbid 22110, Jordan; 2 Department of Computer Engineering, German Jordanian University, P.O. Box 35247, Amman 11180, Jordan

**Keywords:** Wi-Fi, Channel State Information (CSI), Human activity recognition (HAR), Non-line-of-sight (NLOS) environment, Line-of-sight (LOS) environment

## Abstract

The aim of this paper is to present a dataset for Wi-Fi-based human activity recognition. The dataset is comprised of five experiments performed by 30 different subjects in three different indoor environments. The experiments performed in the first two environments are of a line-of-sight (LOS) nature, while the experiments performed in the third environment are of a non-line-of-sight (NLOS) nature. Each subject performed 20 trials for each of the experiments which makes the overall number of recorded trials in the dataset equals to 3000 trials (30 subjects × 5 experiments × 20 trials). To record the data, we used the channel state information (CSI) tool [1] to capture the exchanged Wi-Fi packets between a Wi-Fi transmitter and receiver. The utilized transmitter and receiver are retrofitted with the Intel 5300 network interface card which enabled us to capture the CSI values that are contained in the recorded transmissions. Unlike other publicly available human activity datasets, this dataset provides researchers with the ability to test their developed methodologies on both LOS and NLOS environments, in addition to many different variations of human movements, such as walking, falling, turning, and pen pick up from the ground.

## Specifications Table

**Table:** 

Subject	Computer Science Applications
Specific subject area	Pattern recognition, Human activity recognition based on Wi-Fi signals analysis.
Type of data	Raw dataset, table
How data were acquired	The dataset of Wi-Fi signals was captured in three indoor environments using the CSI tool [1]. Thirty subjects participated in the data collection process. In each of the three environments, the subjects were instructed to perform a set of pre-explained experiments. These experiments were performed in an area located between two desktop computers, each of which is retrofitted with an Intel 5300 network interface card (NIC). One of the two desktop computers was configured to operate as a transmitting device with one antenna, while the other computer was configured to operate as a receiving device with three antennas.
Data format	Raw
Parameters for data collection	The designed experiments were described comprehensively to each of the participating subjects before the start of the experiments. For the first and second environments, a LOS architecture was used. Moreover, a NLOS architecture was used in the third environment. The Intel 5300 NIC was configured to operate at the 2.4 GHz frequency band, wireless channel number 3, channel bandwidth of 20 MHz, and sampling rate of 320 packets/second.
Description of data collection	The data collection process took place in three environments. For each of the three environments, ten subjects performed five different experiments. Each subject performed twenty trials for each of the experiments. The exchanged Wi-Fi signals were recorded, including the RSSI and the CSI values.
Data source location	Institution: Jordan University of Science and Technology, Department of Network Engineering and Security City/Town/Region: Irbid, 22110 Country: Jordan Latitude and longitude (and GPS coordinates) for collected samples/data: 32.4913° N, 35.9875° E
Data accessibility	Repository name: Mendeley Data Data identification number: 10.17632/v38wjmz6f6.1 Direct URL to data: https://data.mendeley.com/datasets/v38wjmz6f6/1

## Value of the Data

• The data were recorded for thirty subjects in three environments in both LOS and NLOS configurations.
• The dataset presented in this paper can be used to assess the performance of Wi-Fi-based HAR systems.
• The collected dataset can be utilized to provide insights on how the different human activity recognition system operates when confronted with activities performed in a NLOS configuration.
• The dataset can also be used by medical alert systems to distinguish a falling incident from other regular daily activities.

## 1 Data Description

The collected raw signals were stored in one main directory that contains three subdirectories. These subdirectories comprise the data that were recorded in the aforementioned three different environments. In each of these subdirectories, the data acquired for 10 different subjects are available. Each subject performed five experiments and repeated each experiment 20 times. The total number of files in the subdirectory associated with each environment is 3000 files. Each file is associated with a specific trial and is stored as a comma-separated values data file (.csv). We divided each experiment to activities as provided by Table 1.

**Table 1:** Description of the fields contained within the structure of the captured Wi-Fi packets

Experiment indicator	Experiment	Activity indicator	Description
C1	Falling from sitting position	A1	Sit still on a chair
		A2	Falling down
		A3	Lie down
C2	Falling from standing position	A4	Stand still
		A5	Falling down
		A3	Lie down
C3	Walking	A6	Walking from transmitter to receiver
		A7	Turning
		A8	Walking from receiver to transmitter
		A9	Turning
C4	Sit down and stand up	A1	Sit still on a chair
		A10	Standing up
		A4	Stand still
		A11	Sitting down
C5	Pick a pen from the ground	A12	Pick a pen from the ground

Each data file is named based on the format “Ex_Sy_Cz_Ai_Tk.csv”, where the description of the data files naming conventions is provided in Table 2.

**Table 2:** Data files naming convention

Symbol	Abbreviation for	Range
E	Environment	{1, 2, 3}
S	Subject	{1, 2, 3, …, 30}
C	Experiment Class	{1, 2, 3, 4, 5}
A	Activity	{1, 2, 3, …, 12}
T	Trial	{1, 2, 3, …, 20}

For example, a data file with the name “E1_S04_C3_A7_T17.csv” refers to data collected in the first environment for subject number 4 during his/her engagement in activity number 7 (turning) of the third experiment (walking between the transmitter and the receiver) and the trial number is 17.

Each collected data file contains a vector of m packets recorded while performing a certain experiment trial. Each of these packets is stored in a row in the csv file associated with the activity trial. The description of each row entry is provided in Table 3.

**Table 3:** Description of the fields contained within the structure of the captured Wi-Fi packets.

Field	Description
timestamp_low	The packet's arrival time that is extracted from the 1MHz NIC clock [1].
bfee-count	The count of the number of beamforming measurements that had been recorded by the CSI tool.
Nrx	The number of the receiving antenna. In our experiments, we are using 3 receiving antennas.
Ntx	The number of the transmitting antennas. In our experiments, we are only using one antenna at the transmitter side.
RSSI_a	The RSSI over the first receiver antenna measured in dB.
RSSI_b	The RSSI over the second receiver antenna measured in dB.
RSSI_c	The RSSI over the third receiver antenna measured in dB.
noise	The existed channel's noise measured in dB.
agc	The automatic gain control that is required to transform the measured RSSI value from dB to dBm.
perm	Refers to the order of the received signals compared to the receiver antennas. For example, [3 1 2] indicates that the first antenna received the signal that belongs to the third RF chain, the second antenna received the signal from the first RF chain, and the third antenna received the signal from the second RF chain.
rate	The rate at which the packets are received measured in packets/second.
CSI	The CSI values are presented as a complex number. In our dataset, we have 90 CSI values associated with each received packet. Each CSI value is indexed as csi_x_y_z where x refers to the transmitting antenna, y refers to the receiving antenna, and z refers to the CSI subcarrier. Since we have only one transmitting antenna, the value of x is always 1. The value of y will be between 1 and 3 since we have three receiving antennas, and the value of z will be between 1 and 30 since for each transmitter-receiver pair we have 30 CSI subcarriers.

**Fig. 1:**
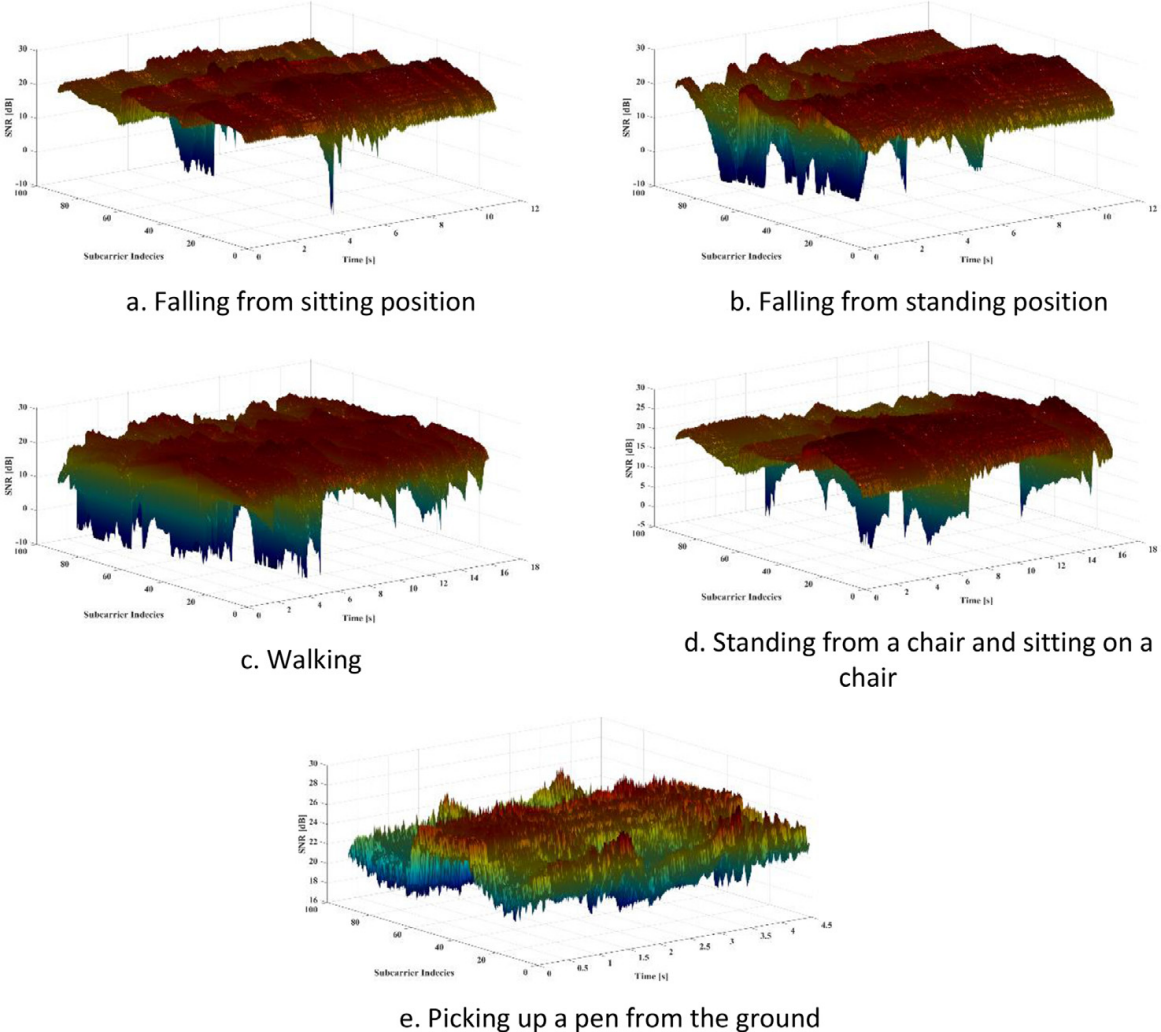
The raw CSI signals recorded during one of the performed experiments. Figure 1.a. Falling from sitting position. Figure 1.b. Falling from standing position. Figure 1.c. Walking. Figure 1.d. Standing from a chair and sitting on a chair. Figure 1.e. Picking up a pen from the ground.

Fig. 1 shows the recorded raw CSI signals for each of the five experiments performed by the first subject in the first environment.

## 2 Experimental Design, Materials and Methods

### 2.1 Subjects

In total, 30 healthy subjects (28 males and two females) have voluntarily participated in the data collection process. The subjects had a mean ± standard deviation age, height, and weight values of 22.7 ± 2.95 years, 178.37 ± 8.4 cm, and 81.9 ± 18.16 Kg, respectively. All participating subjects received a comprehensive description of the experiment that they will perform. Table 4 shows the details of the participating subjects.

**Table 4:** Details of the utilized environments along with the subject's gender, age, weight, and height.

Environment ID	Environment Configuration	Subject ID	Gender	Age (years)	Weight (kg)	Height (cm)
1	LOS	S1	male	35	75	176
		S2	male	21	63	188
		S3	male	25	75	184
		S4	male	23	70	178
		S5	male	21	77	176
		S6	male	20	85	180
		S7	male	21	90	185
		S8	male	21	115	187
		S9	male	21	90	174
		S10	male	28	82	180
2	LOS	S11	male	23	93	180
		S12	male	26	60	178
		S13	male	20	98	188
		S14	male	21	104	172
		S15	male	25	79	175
		S16	male	21	70	182
		S17	male	24	130	185
		S18	female	24	60	160
		S19	male	23	85	175
		S20	female	24	62	153
3	NLOS	S21	male	21	70	180
		S22	male	22	94	186
		S23	male	21	91	192
		S24	male	21	57	173
		S25	male	21	118	167
		S26	male	21	73	178
		S27	male	21	56	173
		S28	male	23	72	171
		S29	male	23	92	188
		S30	male	21	72	187

### 2.2 Experimental procedure

Each subject was asked to perform five experiments. The steps required to successfully perform the experiments were explained before to the beginning of the experiments. To collect several instances of the same experiment, the participating subjects were asked to repeat each experiment for 20 trials.

To ensure accurate performance of the planned experiments, five timing diagrams were designed. These timing diagrams describe how to perform the different experiments and what are the activities involved in each one of them. Furthermore, to inform the subject of when to start a certain activity within the current experiment, a series of programmed beep sounds were used. Particularly, three beep sounds were used as follows:

1 A short beep was used to indicate the start of the experiment trial;
2 A medium beep was used to indicate the end of an activity and the beginning of the next activity; and
3 A long beep was used to indicate the end of the experiment.

Table 5 shows the timing diagrams associated with each of the different experiments. All the numbers on the timing diagrams are in seconds. Before performing the experiments, the timing diagrams were thoroughly explained to the subjects and any question any subject had on the data collection process was answered. Each subject was asked to follow these pre-set timing sequences to have a uniform timing for all subjects who participated in the study.

**Table 5:** The timing diagram for each of the five experiments. A sound icon is used to mark the locations of the added beep sounds.

### 2.3 Software and equipment

**Fig. 2:**
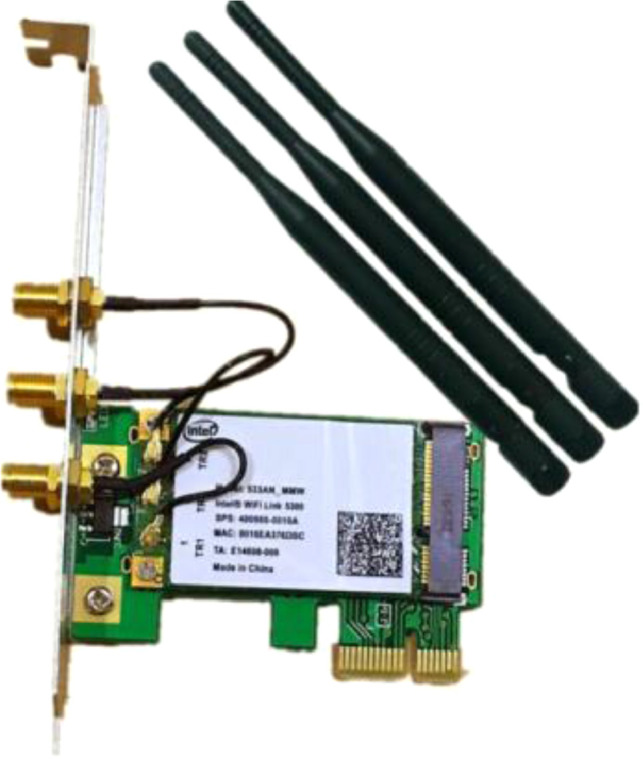
Intel 5300 NIC.

Two desktop computers were equipped with the Intel 5300 NIC to transmit and capture the Wi-Fi packets. The CSI tool [1] was used to capture and process the transmitted packets. Fig. 2 shows the Intel 5300 NIC. Both network cards were configured to operate at the 2.4 GHz band and use the third channel with a channel bandwidth of 20 MHz. The sampling rate was set to 320 Packets/second and the packet size was set to 1 Byte. To transmit the data packets, the injection mode based on LORCON codes [2] is used. Both NICs were configured to operate according to the 802.11n standard, which relies on Orthogonal Frequency Division Multiplexing (OFDM) modulation scheme that enables efficient data transmission over multiple channels [3, 4]. The transmitting NIC is equipped with a single antenna while the receiving antenna is equipped with three receiving antennas that capture the Wi-Fi packets. Using these configurations, a Multi-Input Multi-Output (MIMO) system, which consists of 1 × 3 Wi-Fi streams, was used. The CSI tool used to capture the Wi-Fi signals at the receiving NIC can capture 30 CSI subcarriers for each stream. In other words, we are capturing 3 × 30 CSI subcarriers.

### 2.4 Environment

We collected data in three different environments. For the first and the second environments, we captured the Wi-Fi signals in a LOS configuration. For the third environment, a NLOS configuration was used.

**Fig. 3:**
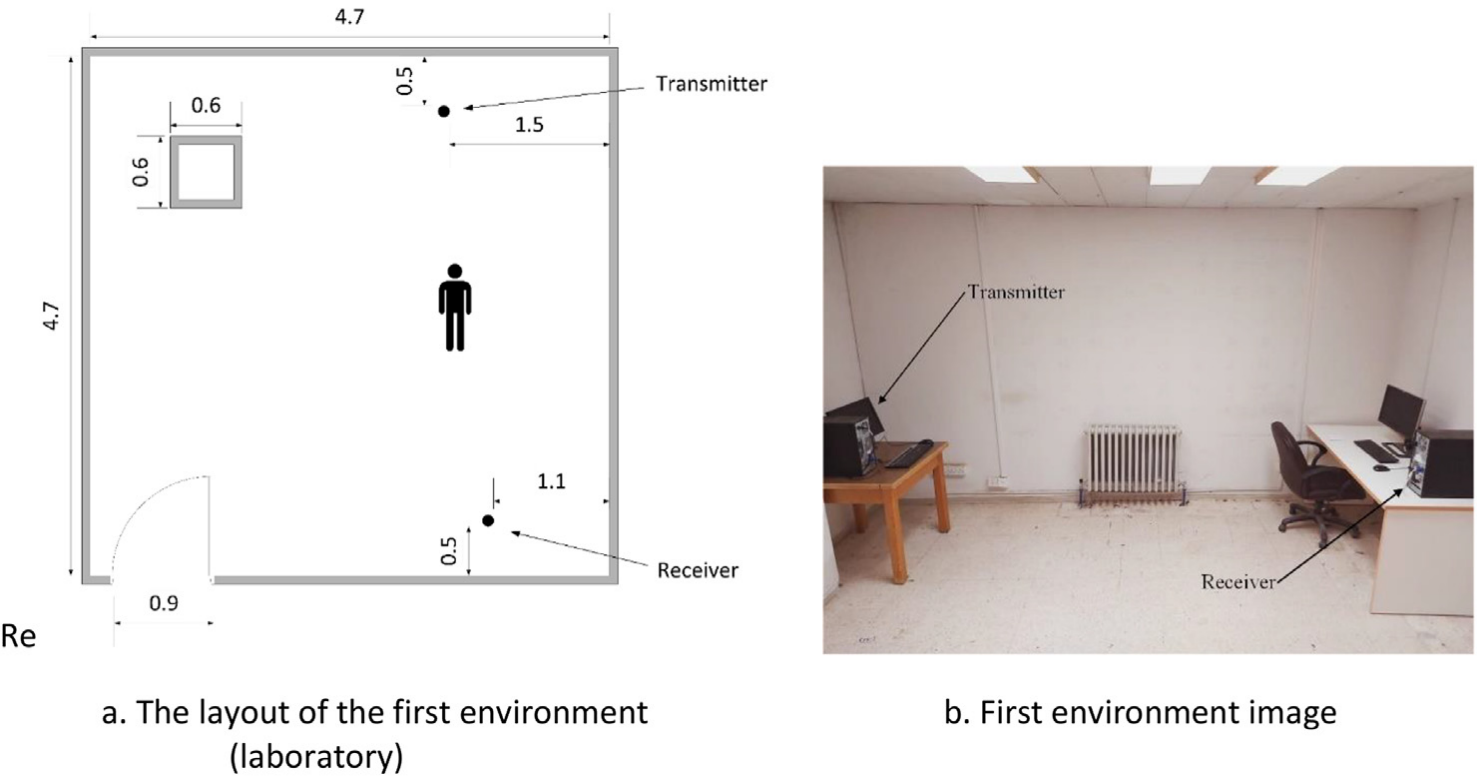
First environment. Figure 3a. The layout of the first environment (laboratory). Figure 3b. First environment image.

In the first environment, we captured the Wi-Fi signals in our research laboratory. The dimensions of the laboratory are 4.7 m × 4.7 m. The transmitter and the receiver were placed at 3.7 m apart from each other. Fig. 3a shows a sketch of the environment, while Fig. 3b shows an image of this environment. All subjects were instructed to perform the pre-explained activities in the middle location between the transmitter and the receiver.

**Fig. 4:**
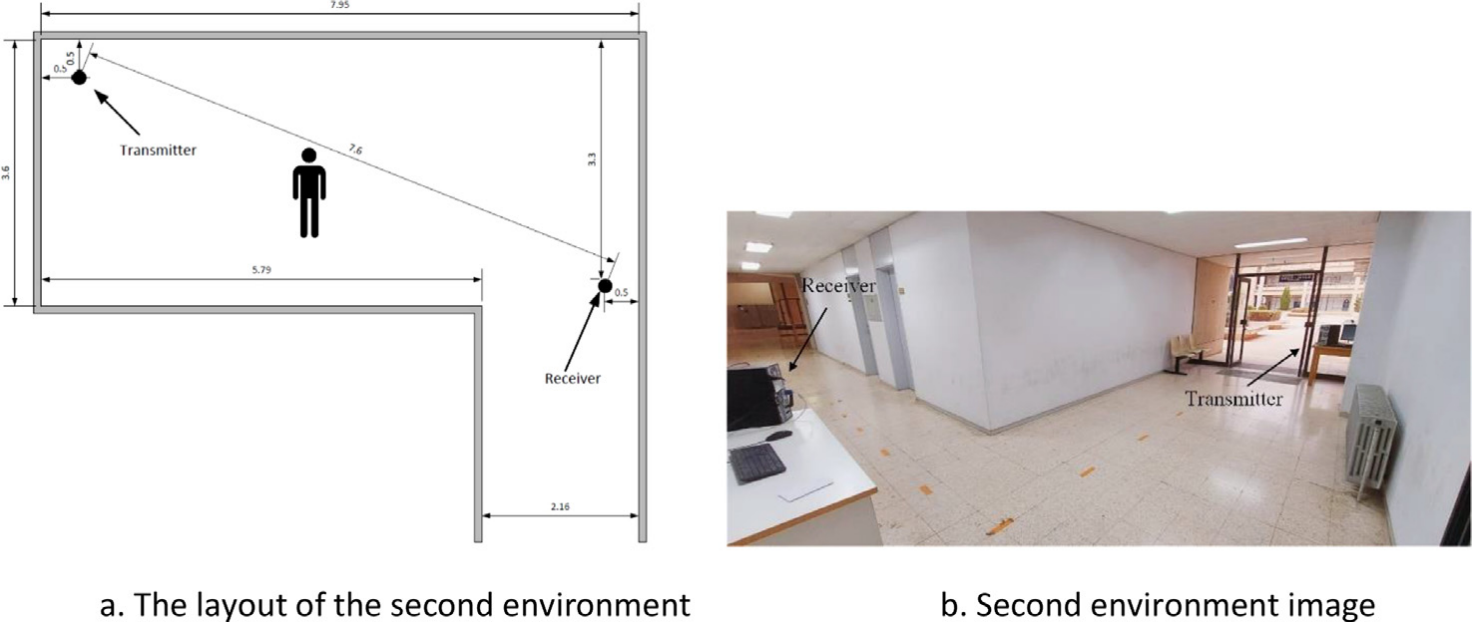
Second environment. Figure 4a. The layout of the second environment. Figure 4b. Second environment image.

In the second environment, the Wi-Fi signals were captured in a university hallway of dimensions 7.95 m × 3.6 m. In this environment, the transmitter and the receiver were placed at 7.6 m apart from each other. Fig. 4a shows a sketch of this environment while Fig. 4b shows an actual image of the environment. As before, the subjects were instructed to perform their pre-explained activities near the centre between the transmitter and the receiver.

**Fig. 5:**
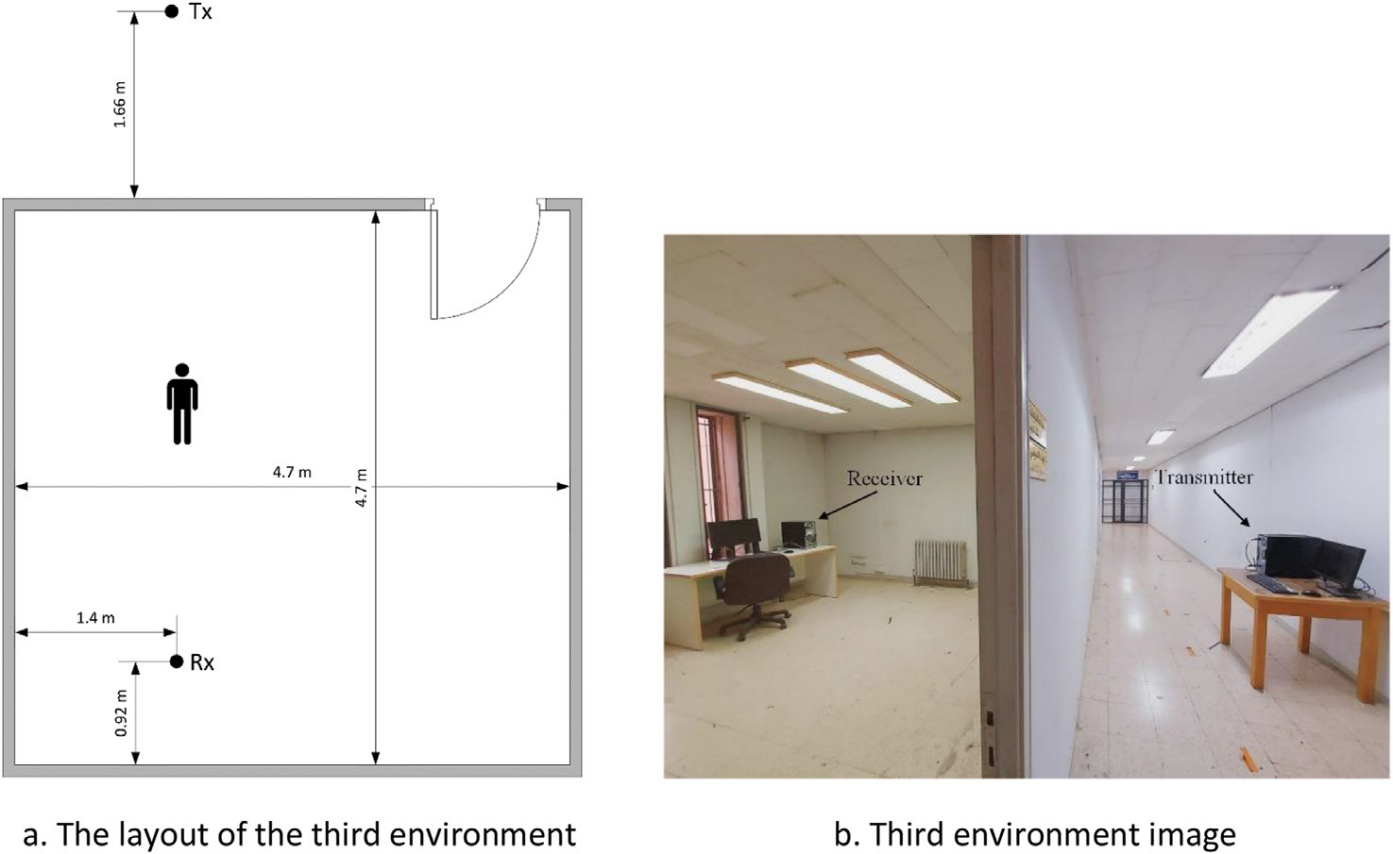
Third environment. Figure 5a. The layout of the third environment. Figure 5b. Third environment image.

The experiments performed in the third environment differ from the experiments carried out in the first and the second environments in the sense that there was a barrier between the subject performing the experiment and the device capturing the Wi-Fi signals. In other words, the transmitter and the receiver were in a NLOS configuration. Specifically, in the third environment, there was a barrier wooden wall with thickness of 8 cm between the transmitter and the receiver. The transmitter was placed outside the room while the receiver was placed inside the room. The distance between the transmitter and receiver was fixed at 5.44 m. An illustration and an image of the third environment is provided in Fig. 5.

## 3 Author Contributions

Conceptualization, B. Alsaify, R. Alazrai; Data Curation, B. Alsaify, M. Almazari; Writing-Original draft preparation, B. Alsaify; Software, M. Almazari; Writing-Reviewing and Editing, B. Alsaify, R. Alazrai, M. Daoud; Validation, B. Alsaify, M. Almazari.

## 4 Ethics Statement

The experimental procedure was performed according to the Declaration of Helsinki and approved by the Institution Review Board (IRB) office at the Jordan University of Science and Technology. Before performing any of the experiments, each subject was asked to sign a consent form in which they were informed that their personal information will not be disclosed and that they have the right to stop participating in any of the experiments at any time if they chose to do so.

## Declaration of Competing Interest

The authors declare that they have no known competing financial interests or personal relationships which have, or could be perceived to have, influenced the work reported in this article.
